# Are we shy of clinical research in India?

**DOI:** 10.4103/0974-2069.58310

**Published:** 2009

**Authors:** Bharat Dalvi

Despite the wealth of clinical material, we as a nation continue to procrastinate when it comes to research. Having managed thousands of cases of rheumatic fever, we still depend upon the West for defining the criteria for diagnosis of rheumatic fever.[1] With the experience of performing hundreds of balloon mitral valvotomies, we continue to rely on the mitral valve scoring system designed on the basis of data obtained from twenty odd patients.[2] This pathos, is not only limited to pediatric or adult cardiology but cuts across the various specialties of medicine and may be even across all sciences. It is therefore worth asking ourselves - why are we averse to clinical research in India? Answers may be uncomfortable and varied opinions are likely on an issue so vital for an upcoming nation.

The problem begins right from our formative years. I remember being reprimanded as a child for touching some inexpensive (unbreakable!) objects at a family friend’s house. The disciplinary intention was honorable, lest I break something and to foster good manners. Similarly, touching toys or clothes in a store was frowned upon. Those who followed rules were considered “good” children and those who dared to break them were labeled “spoilt”. The instinct to explore was nipped in the bud. Schools and teachers followed a similar trend. The curriculum and examination pattern required one to answer questions without challenging the intellect. There was no room for asking smart questions or trying to arrive at smart answers. A curious question or an unorthodox answer was labeled as “being oversmart”. There was no socio-cultural or educational milieu to explore, experiment, innovate or invent. However, I believe current parenting culture is becoming more liberal and schools are changing their curricula and assessments so as to encourage researching at a young age.[3]

Medical schools were not very different. Most professors did not encourage someone trying to work on a problem if it fell outside the realm of the curriculum or the faculty’s knowledge base. If you tried to discuss a novel solution to a problem, most often, you were told “they had tried it all” and there was no point wasting time. Nurturing academic dissidence was unheard of. It was an exceptional teacher who encouraged students to challenge his views. Thus, on completing medical education, if any desire to research was still left, you had to find time. Most clinicians in major teaching and research institutes are so burdened with clinical responsibilities that finding time to design and execute a smart study is almost an impossibility. Funding is hard to come by unless you belong to the magic circle. Remuneration for scientists researching on clinical issues is so paltry that it shoos away smart brains from the profession to a more lucrative clinical practice. Also, with the current economic downturn, sorting out “economics of research” is a greater challenge. The western world too has its own ‘crisis’ in clinical research with a marginally different perspective.[4‐6]

As a society, we make heroes out of warriors, sportsmen, film stars, singers, entertainers, politicians, business tycoons, journalists and even criminals. Rarely have I seen the media make a hero out of a scientist or researcher. A country of over one billion, we merely have half a dozen people winning the Nobel Prize. Of these, half are foreign citizens and have done most of their work on the foreign soil, indicating perhaps that although there is enough talent in the country, the environment is far from perfect.

The current generation apparently seems to be making significant forays in the areas of research, as per a recent report from Thomson Reuters, which states that the research output in India has increased by over 80% in the last 10 years and is expected to overtake majority of the developed countries by the year 2020.[7] However, a closer look at these figures may reveal that most of this output is contributed by clinical trials, designed and executed by foreign multinationals to test their molecules on Indian patients. Is there any “real” research being done - Are new questions being asked or new solutions being sought for old questions? Nevertheless, an important spin-off from this piggyback research is that it will orient our minds and inculcate a discipline required for research.

Genuine research is possibly the only way to survive, sustain and evolve in this competitive world. Solutions to our scenario may sound idealistic and perhaps unrealistic, but do need to be considered. Medical education should be inspiring, there should be compulsory exposure to courses in basic sciences early in the curriculum, with participation in small projects. The medical fraternity needs to work together in cooperative groups or multicentric studies, pooling together available tools and resources for research. Advocacy is vital at the governmental level for creating a milieu to retain talent in academic institutes and also, with private philanthropies for supporting medical research. Finally, a healthy, integrative partnership needs to be fostered between the government, private health care sector and the industry to create a salubrious environment, albeit maintaining the intellectual independence of clinical researchers. We need to invest money, time and talent in research if we are serious about our nation’s future as one of the world leaders of the twenty first century.

To quote Charles Merriam “ The future belongs to those who fuse intelligence with faith and who, with courage and determination, grope their way forward from chance to choice, from blind adaptation to creative evolution”
